# Experimental Infection of Rhesus Macaques and Common Marmosets with a European Strain of West Nile Virus

**DOI:** 10.1371/journal.pntd.0002797

**Published:** 2014-04-17

**Authors:** Babs E. Verstrepen, Zahra Fagrouch, Melanie van Heteren, Hester Buitendijk, Tom Haaksma, Niels Beenhakker, Giorgio Palù, Justin M. Richner, Michael S. Diamond, Willy M. Bogers, Luisa Barzon, Stefan Chabierski, Sebastian Ulbert, Ivanela Kondova, Ernst J. Verschoor

**Affiliations:** 1 Department of Virology, Biomedical Primate Research Centre, Rijswijk, The Netherlands; 2 Animal Science Department, Division of Pathology and Microbiology, Biomedical Primate Research Centre, Rijswijk, The Netherlands; 3 Department of Molecular Medicine, University of Padova, Padova, Italy; 4 Departments of Medicine, Molecular Microbiology, Pathology & Immunology, Washington University School of Medicine, St. Louis, Missouri, United States of America; 5 Department of Immunology, Fraunhofer Institute for Cell Therapy and Immunology, Leipzig, Germany; University of Texas Medical Branch, United States of America

## Abstract

West Nile virus (WNV) is a mosquito-borne flavivirus that infects humans and other mammals. In some cases WNV causes severe neurological disease. During recent years, outbreaks of WNV are increasing in worldwide distribution and novel genetic variants of the virus have been detected. Although a substantial amount of data exists on WNV infections in rodent models, little is known about early events during WNV infection in primates, including humans. To gain a deeper understanding of this process, we performed experimental infections of rhesus macaques and common marmosets with a virulent European WNV strain (WNV-Ita09) and monitored virological, hematological, and biochemical parameters. WNV-Ita09 productively infected both monkey species, with higher replication and wider tissue distribution in common marmosets compared to rhesus macaques. The animals in this study however, did not develop clinical signs of WNV disease, nor showed substantial deviations in clinical laboratory parameters. In both species, the virus induced a rapid CD56^dim^CD16^bright^ natural killer response, followed by IgM and IgG antibody responses. The results of this study show that healthy rhesus macaques and common marmosets are promising animal models to study WNV-Ita09 infection. Both models may be particularly of use to evaluate potential vaccine candidates or to investigate WNV pathogenesis.

## Author Summary

West Nile virus (WNV) is a mosquito-borne virus that can infect mammals, including humans. Most infected humans do not develop disease, but in about 20% of cases humans develop WNV-related disease symptoms, varying in severity from fever to a sometimes life-threatening neuro-invasive disease. The number of WNV infections in Europe has increased in recent years and is caused by viruses that are genetically different from the viruses that caused the WNV epidemic in North America. In this study, we have experimentally infected two different monkey species, rhesus macaques and common marmosets, with the European WNV isolate Ita09 to evaluate the early events after infection and the onset of the disease. Both species were equally susceptible to infection with WNV-Ita09, but differences between species were observed. Compared to rhesus macaques, common marmosets had higher virus loads in blood, and presented a wider distribution of the virus in various organs. Based on the analysis of virological, immunological, biochemical and hematological parameters, we conclude that rhesus macaques as well as common marmosets are potentially useful animal models to evaluate vaccine candidates or to investigate WNV pathogenesis.

## Introduction

West Nile virus (WNV) is a mosquito-borne RNA virus within the Flavivirus genus. Mosquitos of the genus *Culex* are the most important insect vectors, but the virus can also be transmitted by a wide variety of mosquitos from other genera [1]. The enzootic viral lifecycle is between mosquitos and bird species, but transmission can also occur to other hosts, including horses and humans. In humans, the majority of infections (80%) are asymptomatic, and approximately 20% of the infected individuals develop a febrile syndrome, characterized by weakness, myalgia, and fatigue. In less than 1% of WNV-infected patients, illness progresses to a sometimes fatal, neuro-invasive form that results in meningitis, encephalitis, or paralysis. The elderly and immunocompromised are at substantially higher risk for severe WNV disease [2] compared to immunocompentent individuals.

WNV was first isolated in Uganda in 1937 [3], and was originally endemic in Africa, parts of Asia, the Middle East, the southeastern Mediterranean countries, and Australia where a specific subtype of WNV, Kunjin virus, was found [4].

In 1999, WNV was first isolated in New York, and since then has rapidly spread over the American continent. In Europe, the virus sporadically caused infections in horses, but in 1996 an outbreak in Romania caused several human cases of encephalitis. Since then, human fatalities have been reported in Central and Eastern Europe, Greece, and Italy. In addition, surveillance programs have reported equine infections in other Mediterranean countries [5]–[7], suggesting the spread of WNV across the European continent.

Seven phylogenetic WNV lineages have been reported, of which lineages 1 and 2 are the most widely distributed [8]. Until 2004, viruses belonging to lineage 1 caused all WNV infections in Europe, but since then lineage 2 viruses have caused outbreaks in Central and Eastern European countries. In 2010, a lineage 2 virus caused a large epidemic in Greece with 262 human cases [9].

The recent outbreaks of WNV disease in humans [4], [10], [11] highlight an immediate need for a WNV vaccine for human use. Several vaccine candidates for WNV have been evaluated in mice [12],[13], but data obtained from this rodent model have limited prognostic value for the efficacy in humans because of basic differences in the immune system [14]–[16]. Equally, viral pathogenesis and persistence of WNV infection has been studied in mice and hamsters [17]–[22], although the relevance to human disease is not entirely clear. Indeed, WNV infection of inbred rodents is characterized by a rapid development of neurological symptoms and a high mortality rate, features that do not reflect human infection. Non-human primates, with an immune system that is more similar to humans, may provide a more suitable animal model for the development and evaluation of vaccines for use in humans and to study pathogenesis.

Only one vaccine candidate is currently being evaluated in humans [23]–[25]. This candidate is a live attenuated chimeric vaccine, constructed from yellow fever and WNV (pre) membrane genes, making this vaccine less suitable for the population with high risk of developing WNV disease.

Several WNV infection studies in non-human primates have been published. In the 1950s, studies were performed in Old and New World monkey species [26], [27], and more recently, Pogodina *et al.* [28] investigated viral persistence after subcutaneous and intracranial WNV inoculation in rhesus macaques. However, these studies were performed with lineage 1 and 2 WNV strains that pre-dated the recent epidemics that began in the 1990's. More recently, Ratterree *et al.* [29] performed experimental infections of rhesus macaques with the pathogenic WNV NY99 strain, and showed that macaques developed an infection course that was similar to that seen in humans. These results were largely confirmed in a study by Wolf *et al.* [30], who experimentally infected baboons, another Old World primate, with North American lineage 1 isolates of the 2002–2003 epidemics, and that are closely related to the NY99 strain. The NY99 strain has also been used for pre-clinical testing of WNV vaccines in macaques [23], [24], [31]–[33].

Pogodina *et al.* revealed strain-specific variations in the clinical course of infection in macaques [28]. WNV-Ita09 is a strain from Italy, which was isolated during an outbreak in northern Italy in 2008–2009 that caused several human cases of neuro-invasive disease with severe symptoms [34], [35]. WNV-Ita09 and NY99 belong to the same lineage (1a) but to different clades (2 and clade 4, respectively) [8]. Despite genetic similarity of 99.5%, they may have different biological and immunopathological properties. *In vivo* characterization of the European strain WNV-Ita09 is therefore of direct interest to future WNV vaccine development.

As most human infections with WNV are clinically inapparent, little is known regarding the early events of WNV infection. Here, we have developed an infection- model with the WNV-Ita09 strain in rhesus macaques and common marmosets to investigate its replication and tissue distribution. We aimed to investigate the early events using animal species with immune systems that are similar to that of humans. Although the rhesus macaque is an established model for evaluating candidate WNV vaccines, their body-size requires specialized housing and large amounts of test compounds. The development of a smaller and less expensive non-human primate-model, such as the common marmoset, would therefore be of great value. WNV studies using common marmoset have not yet been published, but this species holds promise as it has been proven a suitable animal model for infection with related flaviviruses, such as GBV-B and dengue virus [36]–[40]. Our studies with WNV-Ita09 revealed productive infection of both monkey species, with higher WNV RNA production and wider tissue distribution in marmosets compared to rhesus macaques.

## Methods

### Ethics statement

The rhesus macaques (*Macaca mulatta*) and common marmosets (*Callithrix jacchus*) used in this study were captive bred for research purposes and were socially housed at the Biomedical Primate Research Centre (BPRC) in Rijswijk, The Netherlands. BPRC facilities comply with Dutch law on animal experiments (Wet op de Dierproeven, and its adaptations as published in the Staatscourant), the European Council Directive 86/609/EEC, as well as with the ‘Standard for humane care and use of Laboratory Animals by Foreign institutions’ identification number A5539-01, provided by the Department of Health and Human Services of the United States of America's National Institutes of Health (NIH).

The animals were pair-housed in a BSL3-facility with spacious cages and were provided with commercial food pellets supplemented with appropriate treats. Drinking water was provided *ad libitum*. Enrichment was provided in the form of pieces of wood, mirrors, food puzzles, a variety of food and other home made or commercially available enrichment products. Animals were monitored daily for health and discomfort.

All procedures were approved by the Institutional Animal Care and Use Committee (BPRC Dier Experimenten Commissie, BPRC-DEC; DEC advice #691). The qualification of the members of this committee, including their independence from a research institute, is requested in the Wet op de Dierproeven (1996). At the BPRC all animal handling is performed within the Department of Animal Science (ASD) according to Dutch law. A large experienced staff is available including full time veterinarians and a pathologist. The ASD is regularly inspected by the responsible authority (Voedsel en Waren Autoriteit, VWA) and an independent Animal Welfare Officer. All steps were taken to ameliorate the welfare and to avoid any suffering of the animals. The experimental procedures require anesthesia (surgical insertion of temperature probe) or sedation (intradermal injection of virus, blood sampling). These were performed using ketamine and cepetor (macaques), or Alfaxan and cepetor (marmosets). Monkeys selected for euthanasia were first deeply sedated with ketamine/alfaxan, and subsequently euthanized by intracardiac injection of overdoses pentobarbital.

The Council of the Association for Assessment and Accreditation of Laboratory Animal Care (AAALAC International) has awarded full accreditation to the BPRC. Thus, the BPRC is fully compliant with international demands on animal studies and welfare as set out by the European Convention for the Protection of Vertebrate Animals used for Experimental and other Scientific Purposes, Council of Europe (ETS 123 including the revised Appendix A), Dutch implementing legislation and the Guide for Care and Use of Laboratory Animals.

### Animals

Four common marmosets (*Callithrix jacchus*) and four rhesus macaques (*Macaca mulatta*) were used in this study. All monkeys used in this study were adult animals, ranging in age from 3–4 years old (marmosets), and 8–11 years old (macaques). All animals were purpose-bred and housed at the Biomedical Primate Research Centre (BPRC). The animals were in good physical health with normal baseline biochemical and hematological values. At the start of the study, the animals tested negative for antibodies to WNV. The animals were pair-housed in a BSL3-facility with spacious cages and were provided with commercial food pellets supplemented with appropriate treats. Drinking water was provided *ad libitum*. The experiments were performed in accordance with Dutch law and international guidelines for non-human primates in biomedical research, and were approved by the authorized Institutional Animal Care and Use Committee (BPRC-DEC).

### Experimental infections and post-exposure follow-up

**Figure 1:**
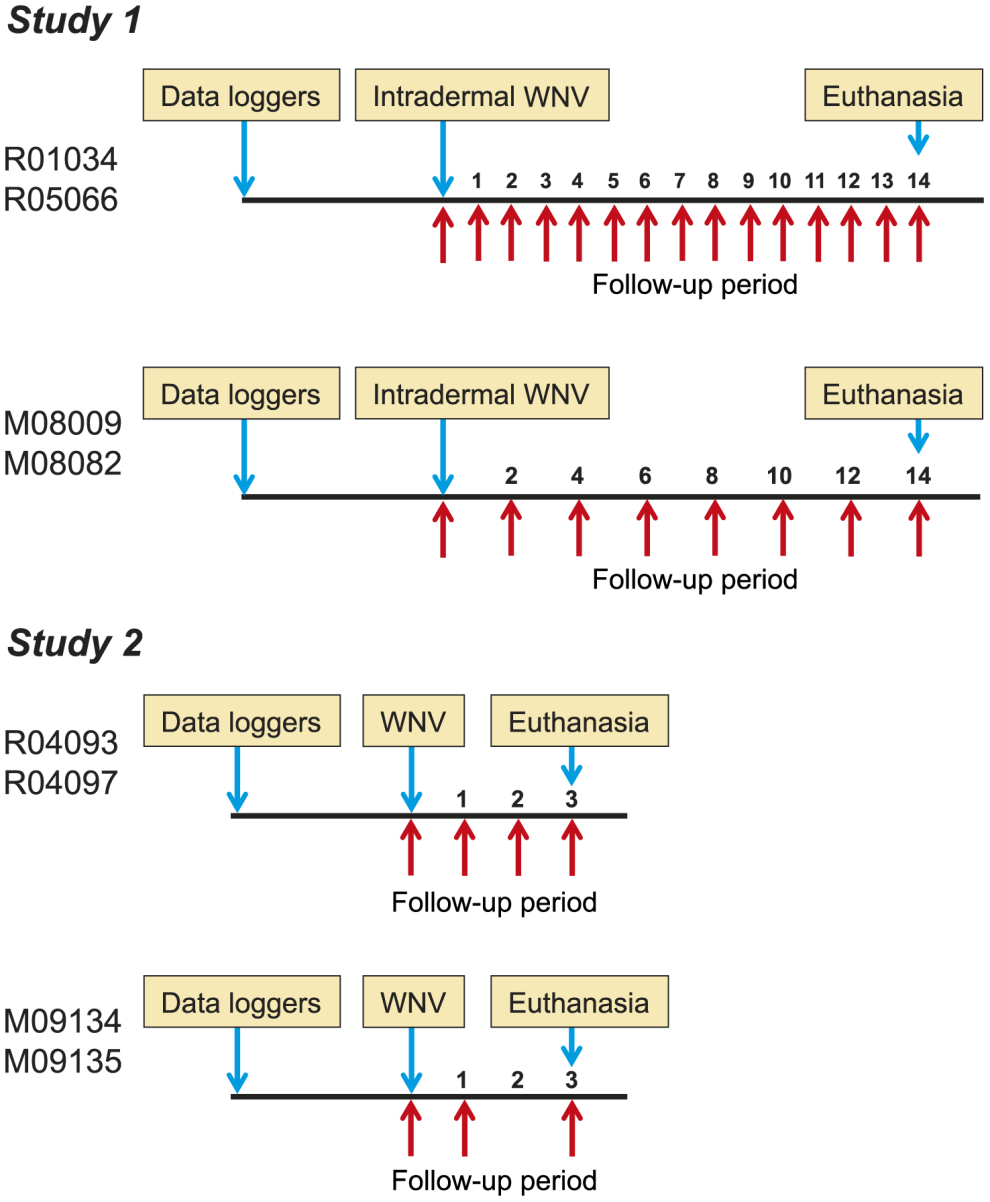
Time schedule. On each time line, blue arrows indicate the time-points of the implantation of the data loggers for temperature registration, the intradermal WNV infection, and euthanasia. Red arrows indicate the bleeding time-points in the follow-up period. The numbers on the time line represent the days post-infection. Names of the animals are depicted with the “R”-animals being rhesus macaques and the “M”-animals being common marmosets.

At day 0, the animals were intradermally inoculated in the upper back with 100 µl of WNV lineage 1a strain Ita09 [35]. Rhesus macaques received 2×10^5^ TCID_50_, a dose that has been described to be infective in rhesus macaques using the NY99 strain [29], [31]. The smaller sized common marmosets received 1×10^5^ TCID_50_ per animal. Virus titration was performed using Vero E6 cells [35]. After WNV infection, the animals were daily observed for general condition, appetite and stool, until the end of the study. Blood was collected using standard aseptic methods from the femoral vein at time points indicated in Figure 1.

A wide range of parameters was analyzed including body temperature and standard hematology and biochemistry. In addition, viral load was quantified in EDTA-plasma, urine and *post mortem* tissue samples. Flow cytometry was performed to assess changes in a wide range of cell populations in peripheral blood.

### Body temperature

Rectal body temperature was measured at time points of sedation using a regular body thermometer. In addition, body temperature of the animals was continuously recorded using DST Micro-T data loggers (UNO Roestvrijstaal BV, Zevenaar, The Netherlands). Fourteen days prior to WNV infection, the thermometer probes were implanted in the abdominal cavity of the animal, and programmed to assess the body temperature every 5 minutes. At euthanasia, the probes were recovered and the collected data were analyzed.

### Urine collection

Pooled urine from pair-housed animals was collected using a tray on the bottom of the cage, and was stored at −80°C until analysis.

### Biochemistry and hematology analysis

A panel of hematological parameters i.e. white blood cell count (WBC), red blood cell count (RBC), hemoglobin, hematocrit, mean cellular volume (MCV), mean corpuscular hemoglobin (MCH), platelets, neutrophils, lymphocytes, monocytes, eosinophils, and basophils, were analyzed in peripheral blood using a Sysmex XT-2000iV Automated Hematology Analyzer (Sysmex Nederland B.V., Etten-Leur, The Netherlands). Biochemical analysis, i.e. creatinine, urea, bilirubin, gamma-glutamyltransferase (GGT), aspartate aminotransferase (AST), alanine aminotransferase (ALT), alkaline phosphatase, lactate dehydrogenase (LDH), iron, albumin, cholesterol, and glucose was assessed using a COBAS Integra 400 plus system (Roche Diagnostics Nederland B.V., Almere, The Netherlands).

### Flow cytometry analysis

To assess changes in lymphocyte subset composition during WNV infection, cells from EDTA-treated blood were stained with seven different antibody combinations, [41] (see: supplementary Table S1). All antibodies have been tested for cross-reactivity with either rhesus macaque or common marmoset cells, and are listed by the NIH Nonhuman Primate Reagent Resource (http://www.nhpreagents.org/NHP/reagentlist.aspx). Immunofluorescence was measured using the FACS Aria (Becton Dickinson BV, Breda, The Netherlands), and data were analyzed with FlowJo software, version 9.6.4 (Tree Star, Stanford University, USA).

### Necropsy

All monkeys were euthanized at designated time points by infusion of pentobarbital (Apharma, Duiven, The Netherlands), and full necropsy was performed. Samples were collected from the following tissues: CNS: cerebellum, hippocampus, brain stem, parietal cortex, and spinal cord (cervical, thoracic, lumbar); oral mucosa, tongue, tonsil, submandibular gland, thyroid gland, bone marrow, esophagus, stomach fundus, pancreas, jejunum, duodenum, ileum, colon, lung, spleen, left and right kidneys, adrenal gland, thymus, inguinal lymph nodes (ing LN), axillary lymph nodes (ax LN), mesenteric lymph nodes (mes LN), heart, urinary bladder, testis or uterus and ovary, skeletal muscle, skin, sciatic nerve, and aorta. All samples were snap frozen for WNV-RNA detection.

### Antibody response

To assess WNV-specific antibodies in plasma, an anti-WNV-E antigen ELISA was performed. Briefly, 96-well microtiter plates were coated overnight with 400 ng of the ectodomain of the WNV-E-protein (NY99 strain), which was expressed in *E. coli* and purified as described previously [42]. Diluted EDTA plasma samples (1∶50) were incubated with the coated antigen for 2 hrs, followed by 1 hr incubation of either goat-anti-human IgG (Thermo Fisher Scientific, Schwerte, Germany) or goat-anti-human IgM (www.antikoerper-online.de, Germany), both HRP-conjugated. After washing, TMB-substrate (BioLegend, Fell, Germany) was added to the wells and the plate was incubated for 30 min at RT in darkness. Then, 1 M H_2_SO_4_ was added to stop the reaction, and plates were measured at 450 nm and 520 nm (reference wavelength) in an ELISA Reader (Infiniti M200, Tecan, The Netherlands).

### Virus detection in blood, urine and tissue

Virus loads in blood were tested with quantitative real-time PCR assay. In addition, a diagnostic nested PCR assay was performed on tissue samples. From each organ collected during necropsy, two or three different tissue pieces were taken for total RNA isolation, and each sample was independently tested for the presence of WNV RNA. Virus loads were determined by real-time quantitative RT-PCR, as described by Lanciotti *et al.* [43]. The lower detection limit of the qRT-PCR was 20 viral RNA copies per reaction. Viral RNA was isolated from plasma and urine samples using a QIAamp Viral RNA Mini kit (Qiagen Benelux BV, Venlo, The Netherlands) following the manufacturer's instructions.

Total RNA was extracted from frozen tissue samples using the RNeasy Plus Mini kit (Qiagen). RNA was reverse-transcribed to cDNA using a Transcriptor First Strand cDNA Synthesis kit (Roche Diagnostics BV, Almere, The Netherlands). WNV was detected using a nested diagnostic PCR assay with an outer primer set WNV-F-out (GAGGACATCTGGTGTGGCAG) and WNV-R-out (ACCTACAGCTTCAGTCAGGC), in combination with an inner set WNV-F-in (TGGTTGAGGACACAGTACTG) and WNV-R-in (TCGCAGACTGCACTCTCCGC). In this test a 345 bp fragment of WNV lineage 1 viruses was amplified. The outer amplification reaction was performed in a 50 µl volume using 10 µl of cDNA, 2,5 units TrueStart Hot Start *Taq* DNA polymerase (Fermentas GMBH, St. Leon-Rot, Germany), 5 µl 10× TrueStart PCR buffer, 1 pmol of each primer, 3 mM MgCl_2_, and 200 µM of each dNTP. In the second amplification reaction, 2 µl of the PCR product of the outer PCR was used as template, and inner PCR conditions were identical to those for the outer PCR. Cycling conditions for both reactions were 95°C for 20 sec, 55°C for 20 sec, and 72°C for 40 sec.

### Virus isolation from plasma

Plasma samples from days 0, 2, 6 and 14 post-infection were used for virus titration on Vero cells. Plasma samples were tested in triplicate in 96-well culture plates that were seeded with Vero cells to 80% confluence. The plasma samples were tested at 2-fold dilutions, starting with a 1∶25 diluted sample. WNV titers were calculated using the method of Reed and Muench [44].

### Statistical analysis

Statistical analysis was performed using Graph Pad Prism version 6.0b.

Difference in total virus production, defined as area under the curve, was analyzed using two-tailed unpaired t-test. A significant difference was defined as p<0.05.

Statistical analysis of the hematology and biochemistry data was performed as follows: the mean value of the two rhesus macaques measured at time point x was compared with the mean value of the two animals at time point 0 using a 2-way ANOVA test. Significant difference was defined as p<0.05.

## Results

### WNV replication in rhesus macaques and common marmosets

The WNV infection study was divided into two experiments. The first experiment was performed to determine the kinetics of viral infection in the two monkey species. In addition, a set of hematological and biochemical parameters was monitored in two rhesus macaques and two common marmosets. *Post mortem* organ examination was performed at two weeks after infection. In the second experiment we determined the virus distribution in various tissues at the peak of WNV infection (Figure 1).

**Figure 2:**
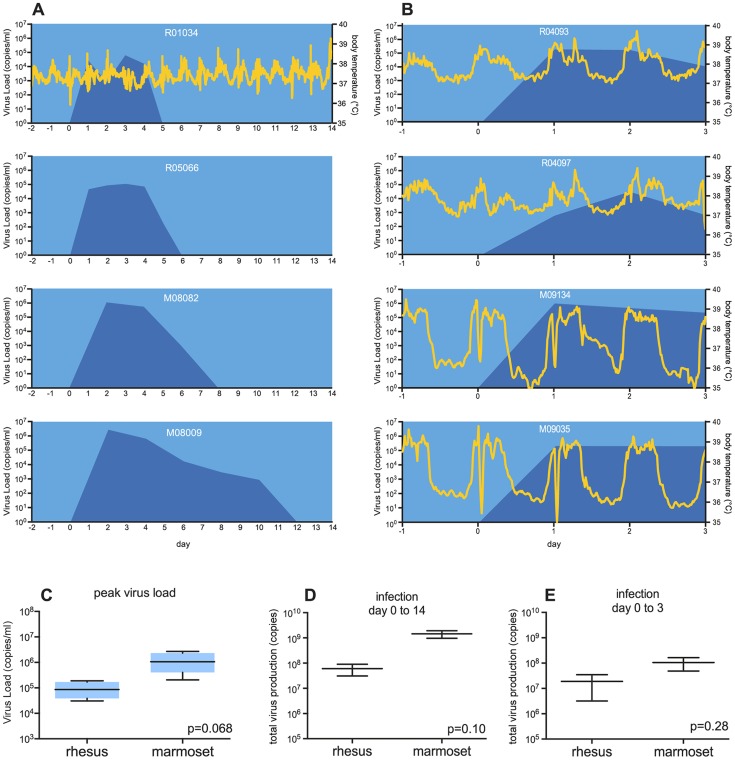
Viral kinetics and body temperature. Viral kinetics and body temperature of animals during (A) 14-days follow-up, and (B) 3-days follow-up. Dark blue areas represent the quantification of the WNV RNA load (RNA copies per ml, plotted on the left y-axis). The yellow lines represent the body temperature (in °C on the right Y-axis). Both parameters are plotted against time in days (x-axis). Due to technical failure of the temperature registration software only the body temperature registration of R01034 could be retrieved from the data logger. (C) Peak virus load was calculated for rhesus macaques and compared with the peak virus load of marmosets. (D) Total virus production in rhesus macaques and marmosets from the 14-days infection study calculated from the area under the curve (AUC). (E) Total virus production in rhesus macaques and marmosets from the 3-days infection study calculated from the AUC.

Intradermal inoculation with the WNV strain Ita09 was successful in both monkey species, as was reflected by the viremia that was detectable as early as one day after inoculation (Figures 2A and B; dark blue area). Marmosets showed a longer viremic period compared to the rhesus macaques (8 to 10 days versus 4 to 5 days). Marmosets also developed higher peak virus loads than rhesus macaques 1.3×10^6^ and 9.9×10^4^ copies of WNV RNA/ml plasma, respectively (Figure 2C) that trended towards statistical significance (*P* = 0.068). The area under the curve (AUC) was used to calculate the total virus production during viremia. When this was calculated for each individual animal and was compared between the species, marmosets had produced more viral RNA, both over the entire viremic period (1.4×10^9^ compared to 6.0×10^7^ copies/ml plasma; Figure 2D), as well as in the first three days of infection (1.0×10^8^ compared to 2.0×10^7^ copies/ml plasma; Figure 2E). Although higher virus production was measured in common marmosets compared to rhesus macaques, statistical significance was not reached, likely due to the small group sizes.

Because viral RNA detection using qRT-PCR does not differentiate between replicating-competent and defective viruses, we performed virus isolation on plasma samples taken at 0, 2, 6, and 14 days post-infection from the marmosets and macaques of the 14-days infection experiment. In plasma collected at day 2 p.i., which is at the peak of viral RNA production, marmosets M08009 and M08082 presented a plasma virus titer of 3249 and 2378 TCID_50_/ml, respectively, while the infectious virus titers in macaques R01034 and R05066 were 354 and 119 TCID_50_/ml, respectively. Thus, in both species 2–3 log more viral RNA was detected using qRT-PCR than infectious virus particle titers measured by titration on Vero cells. Virus isolation from all other time-points was negative.

In humans, WNV was detected in urine for as long as one month post-infection [45], although this finding remains somewhat controversial [46], [47]. To assess if WNV could be detected in urine from monkeys shortly after infection, RT-PCR was performed on RNA isolated from pooled urine samples from the macaques. No evidence was found that WNV was secreted in urine of the macaques (data not shown).

### Absence of WNV-induced clinical signs in rhesus macaques and common marmosets

In both studies, none of the animals showed any behavioral changes indicative for neurological illness. Additionally, no increase of body temperature was observed in any of the animals. Only normal variations in body temperature, due to circadian rhythm and/or sedation, were recorded by the data loggers (Figures 2A and B; yellow lines) or were measured with a conventional thermometer.

### Biochemistry and hematological parameters after WNV infection

Biochemistry and hematology data were collected from the animals at days 0, 4, 6, 8 and at the time-point of euthanasia (Supplementary figures S1 and S2).

As impaired kidney function after WNV infection has been described in humans, the transient increase of urea and alkaline phosphatase levels that was observed in plasma of the common marmosets is interesting. However, as creatinine levels were normal and albumin levels were stable, these findings most likely do not reflect significant or sustained renal injury due to WNV infection. Furthermore, as in macaques a temporary increase of the liver enzyme ALT, in combination with a transient increase of alkaline phosphatase and LDH was observed, it rather suggests a transient and mild congestion of the bile ducts. Some minor changes were observed in the hematological parameters in some of the individual animals, but no statistical difference was observed and values remained within normal variation seen in rhesus macaques. In the smaller marmosets, hematocrit showed a statistical significant deviation from the baseline value that is most likely the result of the frequent blood withdrawal during the study.

### Humoral immune response after WNV infection

**Figure 3:**
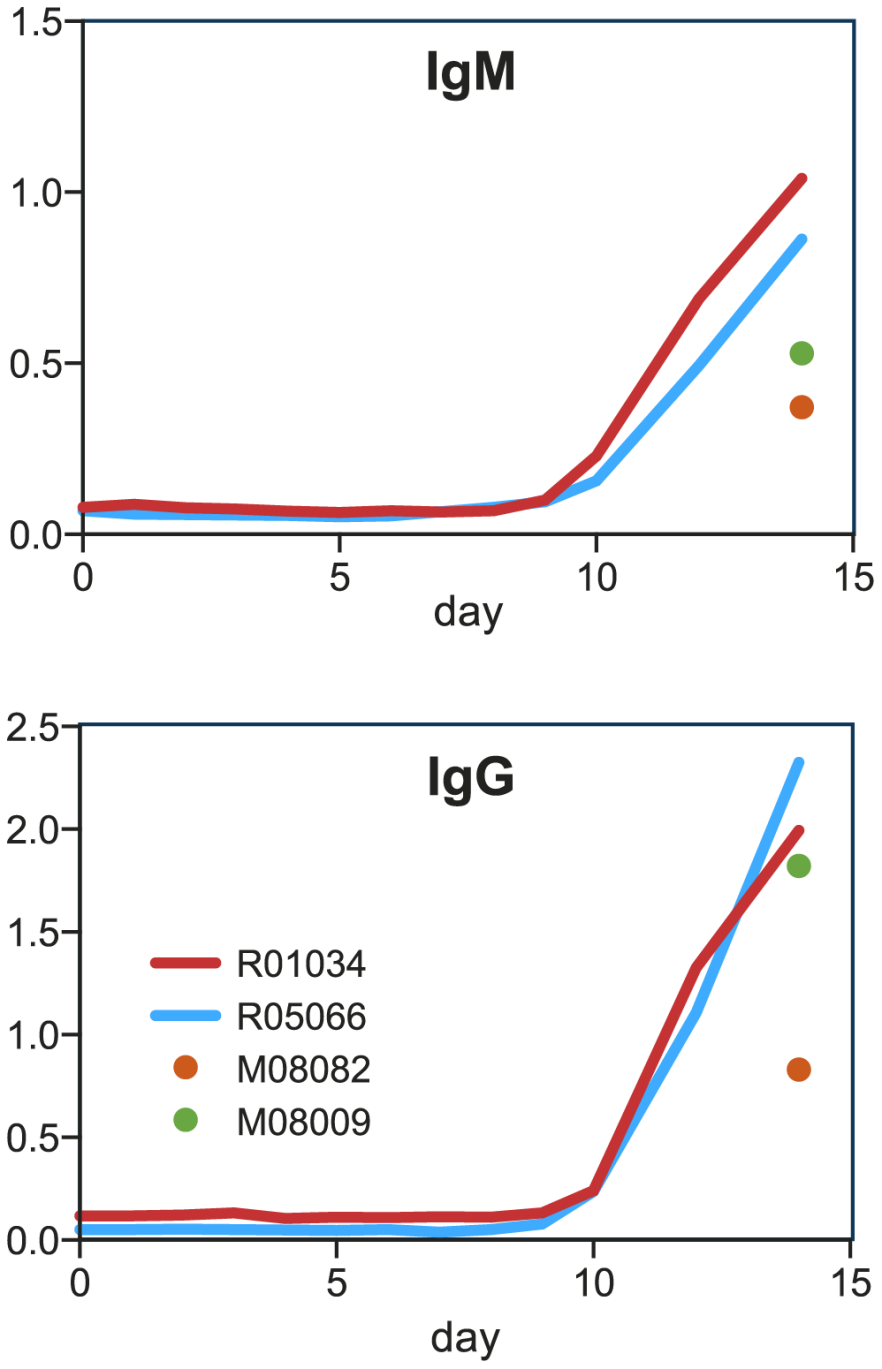
Humoral response after WNV infection. WNV-E-protein-specific IgM and IgG levels of the individual animals during WNV infection. The antibody binding was calculated as the absorbance at 450 nm minus the absorbance at 520 nm. The mean value of two independent measurements of 1∶50 diluted samples is depicted in the figure.

We analyzed sequential plasma samples from the rhesus macaques R01034 and R05066 (14-days infection experiment) for the presence of an anti-WNV E-protein antibody response [48]. Anti-WNV IgM was first detected 9 days post-infection with WNV-Ita09, and titers further increased at days 12 and 14 after challenge (Figure 3). Anti-WNV IgG was detectable at 10 days post-infection, and similar to IgM, IgG levels increased over time. Limited blood sampling from marmosets allowed the determination of antibody levels only at the start of the study and at the time point of euthanasia at day 14. At the start of the study the marmosets were negative for E-antigen-specific IgM and IgG, but both isotypes were clearly detectable at the time point of euthanasia (Figure 3).

### Natural killer cell activation after WNV infection

Because it is still unclear what lymphocyte population is influenced during the first phase of WNV infection, flow cytometric analysis was performed at defined time points. Not only lymphocyte subset compositions were determined, but also T-cell subsets were defined for the expression of proliferation, differentiation, and activation markers. In addition, natural killer (NK) cell, B-cell and dendritic cell subsets were characterized.

T-cells were characterized as CD3^+^ lymphocytes and according to the expression of CD4 and CD8 divided into different subsets (Supplementary figure S3). The markers CD28, CD95, CCR7, CD27CD45RA were then used to determine the activation and differentiation status of the T-cell subsets into naive (CD28+ CD95−), central memory (CM; CD28+ CD95+ CCR7+, CD27+), transitional memory (TM; CD28+ CD95+ CCR7+, CD27−), effector memory (EM; CD28−, CD45RA−), and effector cells (CD28−, CD45RA+). No evidence was found for changes in these T-cell subsets due to WNV infection in any of the animals (Supplementary figure S4).

Next, the B-cells were analyzed. Due to lack of cross-reactivity between some of the antibodies and marmoset lymphocytes, this analysis was only performed on PBMC from rhesus macaques. Cells positive for CD19 and HLA-DR were selected from the lymphogate and divided into three major subsets, CD10^+^, CD10^−^ and IgG^+^ B-cells. CD10^−^ B-cells were further subdivided into plasmablasts (CD27^high^CD21^−^), activated memory B-cells (CD27^dim^CD21^−^), tissue B-cells (CD27^low^CD21^−^), naive B-cells (CD27^low^CD21^+^), and memory B-cells (CD27^low^CD21^+^) (Supplementary figure S5). In none of the monkeys we observed a clear effect of WNV infection on B-cell subsets during the first 14 days of infection.

For the same reason as mentioned above, only the dendritic cells (DC) of the macaques were analyzed. CD3^−^HLA-DR^+^CD20^−^ cells within the lymphogate were analyzed for expression of CD14. CD14^+^ were defined as monocytes, while the CD14^−^ cells were further subdivided into B-cells (CD20+), CD1c^+^ myeloid DC (mDC; CD123^−^CD1c^+^), CD11c^+^ mDC (CD123^−^CD11c^+^) and plasmacytoid DC (pDC; CD123^+^CD11c^−^). No deviations were observed as an effect of WNV infection in rhesus macaques during the first 14 days (Supplementary figure S6).

**Figure 4:**
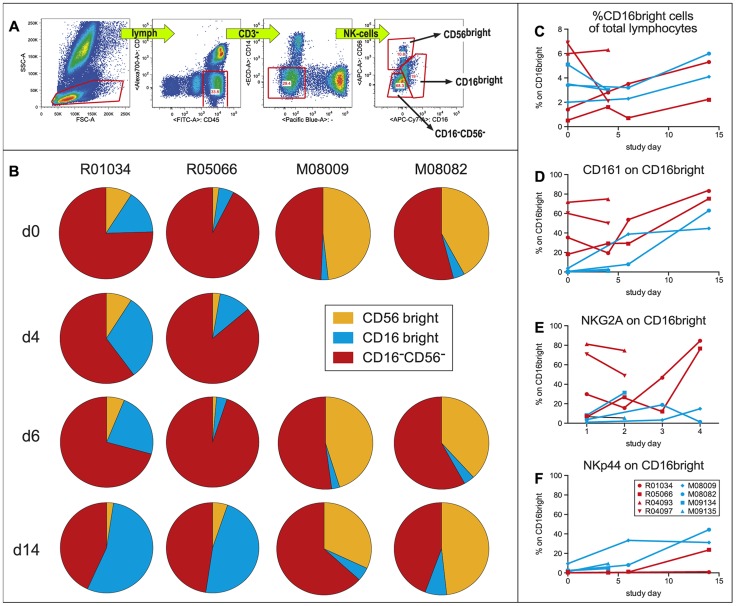
Characterization of NK subsets during WNV infection. (A) Representative example of the gating strategy. CD3−, CD45+, CD14− cells were selected from the lymphogate. Depending on the expression of CD56 and CD16 NK-cells were divided into three subsets. (B) Full circles represent the total NK population and from each individual animal the fraction of CD56^bright^, CD16^bright^ and D16^neg^CD56^neg^ of total NK population is shown at 4 time points after WNV infection. (C) Percentage of CD16^bright^ NK-cells of total lymphocyte population. (D) CD161 expression on CD16^bright^ NK-cells. (E) NKG2A expression on CD16^bright^ NK-cells. (F) NKp44 expression on CD16^bright^ NK-cells.

Because innate immunity, and especially natural killer (NK) cells, plays an important role against WNV infection [49]–[51], we also characterized different NK-cell subsets during the first 14 days of WNV infection. CD3^−^CD45^+^ cells were selected for the absence of CD14 and CD20 surface markers. Based on the expression of CD16 and CD56 within this population, three subpopulations could be distinguished: CD56^bright^, CD16^bright^ and CD56^−^/CD16^−^ double-negative cells (Figure 4A). A general increase of CD16^bright^ cells within the NK-cell population was observed over time in all four monkeys (Figure 4B). This rise in CD16^bright^ cells was not only detectable in the NK-cell fraction, but also in the total number of lymphocytes (Figure 4C). The CD16^bright^ cells were further analyzed for the expression of CD161, NKG2A and NKp44 surface markers. After WNV infection, the expression of CD161, NKG2A, and NKp44 on the CD16^bright^ NK-cells was upregulated (Figure 4D to F). No such effect was observed in other NK-cell subsets.

### WNV distribution in post mortem tissues

Samples from different organs and tissues were collected at necropsy, and analyzed for the presence of WNV RNA by using a diagnostic nested RT-PCR assay. From each organ or tissue, at least 2 pieces of tissue were analyzed in 2 separate tests. In cases when the PCRs were inconclusive, a third piece of tissue was analyzed. The PCR data are summarized in Table 1.

**Table 1:** West Nile virus tissue distribution in infected rhesus macaques and common marmosets.

Day	Animal	Tissue^1^	Sample 1	Sample 2	Sample 3
3	R04097	Spleen	−	+	ND
3	M09134	Ax LN	−	+	no tissue left
		Mes LN	−	+	no tissue left
		Lung	−	−	+
		Adrenal gland	+	+	ND
		Tonsil	+	+	ND
		Gall bladder	+	+	ND
		Trachea	+	+	ND
3	M09135	Ax LN	+	+	ND
		Mes LN	−	+	no tissue left
		Lung	+	−	−
		Adrenal gland	+	+	ND
		Thymus	+	−	+
		Ileum	+	+	−
		Stomach	+	+	ND
		Kidney R	+	−	+
		Urinary bladder	+	−	−
14	R01034	Spleen	+	+	ND
		Ax LN	+	+	ND
		Ing LN	+	−	+
		Urinary bladder	+	−	−
14	R05066	Spleen	+	+	ND
		Ax LN	+	+	ND
		Cerebellum	+	−	−
		Hippocampus	−	+	−
14	M08009	Ax LN	+	+	+
		Ing LN	+	−	+
		Mes LN	+	−	+
		Hippocampus	+	−	−
14	M08082	Spleen	+	−	−
		Ax LN	−	+	no tissue left
		Ing LN	+	ND	no tissue left
		Kidney R	+	−	−

1 For abbreviations see methods section.

Three days after infection more tissues were positive for WNV RNA in the common marmosets than in the macaques. Seven samples were WNV-positive in common marmoset M09134, while in marmoset M09135 nine tissues tested positive. Viral RNA was found in the axillary and mesenteric lymph nodes, the lung, and the adrenal gland of both animals. Samples from the tonsil, gall bladder, trachea, thymus, ileum, stomach, kidney, and urinary bladder were tested positive in at least one of the marmosets. In sharp contrast, only one rhesus macaque (R04097) tested positive in the spleen, while in the other macaque (R04093) no virus was detected in any of the solid tissue samples. Fourteen days post-infection the number of WNV-positive tissues was comparable in both monkey species. West Nile virus RNA was primarily detected in spleen and lymph node samples from both species. In addition, viral RNA was found in the urinary bladder of macaque R01034, and in brain tissues (cerebellum, hippocampus) of macaque R05066. Marmoset M08009 also tested positive in the hippocampus, and M08082 was positive in one of the kidneys.

## Discussion

In this study, we monitored the infection of rhesus macaques and common marmosets after intradermal inoculation with the WNV-Ita09 strain over the first 14 days. Both monkey species tested here were equally susceptible to WNV-Ita09. Common marmosets, however, showed higher viremia and broader tissue distribution as compared to rhesus macaques.

Duration of viremia and time-point of the peak in viral RNA production in the macaques were comparable between the WNV-Ita09 WNV strain and the earlier described infections with the NY99 WNV strain [29]. Ratterree *et al.* reported a transient and low-level viremia that not exceeded 100 TCID_50_/ml. In our study macaques show titers of 119 and 354 TCID_50_/ml, while the titers in marmosets reached 2378 and 3249 TCID_50_/ml. Whether this is an indication of a slightly higher pathogenicity of the Ita09 strain is unclear, as a direct comparison between virus production was not possible because of the different cell types used to quantitate virus levels: the monkey kidney Vero cell line versus titration on the mosquito C6/36 cell line. Using the European strain WNV-Ita09, IgM and IgG titers were detectable at 9 and 10 days after infection, respectively, which is earlier as compared to NY99. WNV-Ita09 induced IgM and IgG with almost similar kinetics, whereas macaques infected with the NY99 failed to develop IgG titers before 3 weeks post-infection [29]. In marmosets, the WNV infection was more pronounced than in the macaques. In both species, viremia peaked at 2–3 days after exposure, but marmosets accumulated a higher virus load, and remained viremic for a longer time period. Despite the high level viremia, none of the animals used in this study showed any apparent clinical symptoms, which is similar to the study performed with WNV NY99. This is not entirely unexpected as the monkeys that were used in both studies were healthy animals. In humans, severe WNV disease occurs only in a small percentage of infections, and is often associated with pre-existing immune disorders [52]. Monkeys with underlying immune disorders can develop WNV disease as exemplified by the fatal infection of an aged Barbary macaque (*Macaca sylvanus*) in the Toronto Zoo [53]. To study the onset of WNV disease, but also to investigate vaccination strategies to be used human target populations, it may be essential to use immunocompromised monkeys in future experiments. In a recent publication, Pham *et al.* [54] used immunocompromised macaques to examine the pathogenicity of pandemic H1N1 influenza A virus showing the validity of such an approach. Equally, others have used similar methods to study measles virus-induced pathogenesis [55], [56], or to set up a macaque model for Epstein-Barr virus pathogenicity in humans [57]. Based on the expression of CD56 and CD16, NK-cells can be divided into different subsets, where CD56^bright^ cells have more immune-regulatory properties, while CD16^bright^ cells are more cytolytic effector cells. Following experimental WNV infection we observed that CD56^dim^CD16^bright^ NK-cells were affected in all examined animals. Though only a limited number of animals (n = 4) were included in this study, the increase in this particular NK population was evident. A higher expression of activating receptors CD161 and NKG2A on CD56^dim^CD16^bright^ NK-cells has been associated with an increased lytic capacity of the NK-cells [58]. Recruitment of CD56^dim^CD16^bright^ NK-cells by macaques in reaction to WNV infection may be a mechanism to control further spread of the virus. A potential role for NK-cells is consistent with the decrease in viremia before an apparent role of the adaptive immune system, either antibodies or T-cells was observed. However, Yoshida *et al.* [59] published that CD16 depletion in tamarins, a New World monkey that is closely related to common marmosets, prior to dengue virus infection had no effect on virus loads, suggesting that NK-cells play only a limited role in the defense against primary dengue virus infection. Similarly, NK-cell depletion in mice had no effect on WNV pathogenesis [60]. Clearly, further investigation is required to define the possible antiviral function of NK-cell subsets in the protective immune response against WNV infection in monkeys and humans.

WNV tissue distribution at three days post-infection was different in marmosets and macaques. After intradermal inoculation the virus rapidly spread to several organs in the marmosets whereas in macaques the viral distribution was limited to blood, and in one animal to the spleen. Fourteen days after infection differences in virus distribution between the species had practically disappeared. The exact cause is unknown; virus dose used to inoculate the monkeys may have influenced tissue distribution as marmosets were infected with a higher dose per kg body weight than macaques. Furthermore, species differences also may have influenced tissue distribution, similar to WNV kinetics and production. Pogodina *et al.* [28] also investigated tissues from rhesus macaques 2 weeks post-infection., and cultured WNV from brain and spinal cord (5 out of 8 monkeys), and from homogenates of internal organs (9% of isolations). However, all animals from the Pogodina study died of fatal encephalitis after cerebral inoculation with a different strain of WNV, which makes a direct comparison between studies not possible.

In the present study West Nile virus RNA was frequently detected in primary and secondary lymphoid tissues, including lymph nodes, tonsils, thymus and spleen. This distribution pattern implies that these tissues represent sites for virus replication; although detection of virus as part of formed immune complexes cannot be ruled out. The latter situation may be less conceivable at 3 days post-infection when no anti-WNV antibodies were detectable, but immune-complexes may have been formed 2 weeks after infection when antibodies were readily found in serum.

West Nile virus can cause serious neurological disorders in humans, and the infection of brain tissues, like the hippocampus (R05066 and M08009) and the cerebellum (R05066), is thus of interest. Beyond this, organs from the endocrine system, like the adrenal gland, the pancreas, and the stomach were also found positive for WNV. The virus has also been documented by others in monkey brain tissues including cerebral cortex, subcortical ganglia, and cerebellum of persistently-infected macaques, irrespective of disease, in addition to kidneys, spleen, and lymph nodes [28]. The combined findings in monkeys resemble WNV distribution in humans as was described in the study of post mortem tissues of six patients that died of WNV infection [52]. The presence of WNV in kidneys of macaques and marmosets is interesting because in humans WNV RNA reportedly is excreted in urine during acute infection [45], [61], [62], although this finding is not fully accepted [46], [47], [63]. Here, we were unsuccessful in retrieving WNV RNA from urine samples from macaques, but we did detect WNV RNA in kidney tissue from common marmosets. We did however detect WNV RNA in urinary bladder samples from two animals. This is an interesting finding since the origin of excreted WNV in urine is unknown and resident kidney cells do not seem permissive for WNV replication [64]. Experiments are ongoing to confirm these findings using immunohistochemistry.

Until now, rhesus macaques and baboons have been the only non-human primate models to study WNV infection and to evaluate potential vaccine candidates. However, research with both species is expensive because of specialized housing and care, and can require large amounts of test compounds because of the size of the animals. A smaller non-human primate model to study WNV infection is therefore desirable. We found that the common marmoset is at least equivalently if not more susceptible to infection with WNV than macaques, and thus may be a valuable asset to WNV research, due to its smaller size and comparable immune response to other primates and humans.

## Supporting Information

Figure S1 **Serum chemistry data.** Changes in biochemistry parameters of the individual rhesus macaques (red and blue lines) and common marmosets (orange and green lines) during the 14-days follow-up period. Day 0 indicates the day of WNV infection. The mean value of the two rhesus macaques measured at time point x was compared with the mean value of the two rhesus macaques at time point 0 using a 2-way ANOVA test. Statistical difference was defined as p<0.05 (represented by * for rhesus macaques). Same method was used to calculate statistical difference in common marmoset samples and this is represented by the # in graphs.
(TIF)

Figure S2 **Hematology data.** Changes in hematology parameters during the 14-days follow-up period of the in individual rhesus macaques (red and blue lines) and common marmosets (orange and green lines). Day 0 indicates the day of WNV infection. The mean value of the two rhesus macaques measured at time point x was compared with the mean value of the two rhesus macaques at time point 0 using a 2-way ANOVA test. Statistical difference was defined as p<0.05 (represented by * for rhesus macaques). Same method was used to calculate statistical difference in common marmoset samples and this is represented by the # in graphs.
(TIF)

Figure S3 **Proliferative T-cells in PBMC after WNV infection.** (A) A representative example of the gating strategy. Cells in the lymphogate were selected based on the expression of CD3. CD3^+^ cells were divided into CD4^+^, CD8^+^, or CD4^+^CD8^+^ double-expressing T-cells. Next, Ki67 expression was determined as a measure for the proliferative properties of the different T-cell populations. (B) Full circle represents the total T-cell fraction per animal per time-point. Within this fraction the CD4^+^ (blue), CD8^+^ (grey), and CD4^+^CD8^+^ (red) T-cells are indicated. The exploded slices from the pie represent the share of proliferating cells per subtype.
(TIFF)

Figure S4 **T-cell differentiation during WNV infection.** (A) Representative example of the gating strategy. CD3 positive cells within the lymphogate were divided into three different populations based on the expression of CD4 and CD8. CD4 T-cells, CD8 T-cells, and double-positive T-cells were divided into naive (CD28^+^CD95^−^), memory (CD28^+^CD95^+^), and effector cells (CD28^−^). Next, based on the expression of CCR7 and CD27, memory cells were divided into central memory (CM) (CCR7^+^CD27^+^) and transitional memory (TM) (CCR7^−^CD27^+^) cells. Effector cells were analyzed for the expression of CD45RA, and split into effector memory cells (EM: CD45RA^−^) and effector cells (CD45RA^+^). (B) Pie diagrams of the differentiation status of CD4^+^, CD8^+^, and double-positive T-cells. Full circles represent the total CD4^+^, CD8^+^, or CD4^+^CD8^+^ T-cell populations in each animal at different time-points post-infection.
(TIFF)

Figure S5 **B-cell subsets during WNV infection in macaques.** (A) Representative example of the gating strategy. CD19^+^HLA-DR^+^ cells were divided into CD10^+^ and CD10^−^ cells. Next, based on the expression of CD27 and CD21, CD10 negative B-cells were divided into plasmablasts, memory B-cells, naive B-cells, activated memory B-cells, and tissue memory B-cells. The percentage of IgG-producing the total number of B-cells was analyzed. (B) The full circles represent the total number of B-cells of the individual animals at different time points while the pie parts represent different B-cell subsets.
(TIFF)

Figure S6 **Dendritic cell subsets during WNV infection in rhesus macaques.** (A) Representative example of the gating strategy for the determination of the different DC subsets. CD3−, CD8−, HLA-DR^+^ cells were selected from the lymphogate. Cells negative for CD20 were analyzed for the expression of CD14. CD14^+^ cells were defined as activated monocytes while CD14^−^ cells were divided into CD1c mDC (CD123^−^CD1^+^), pDC (CD123^+^CD11^−^) and CD11c mDC (CD123^−^CD11^+^). (B) The full circles represent the total number of CD3^−^HLA-DR^+^D20^−^ dendritic cells while the pie parts represent the different DC subsets at the different time points.
(TIFF)

Table S1 **FACS antibodies used in study.** List of antibodies used to assess changes in lymphocyte subset composition during WNV infection.
(DOCX)
