# Supplementary figures and images for: Experimental Infection of Rhesus Macaques and Common Marmosets with a European Strain of West Nile Virus
Source: PLoS Negl Trop Dis. 2014 Apr 17;8(4):e2797. doi: 10.1371/journal.pntd.0002797 (PMC3990483; doi:10.1371/journal.pntd.0002797)

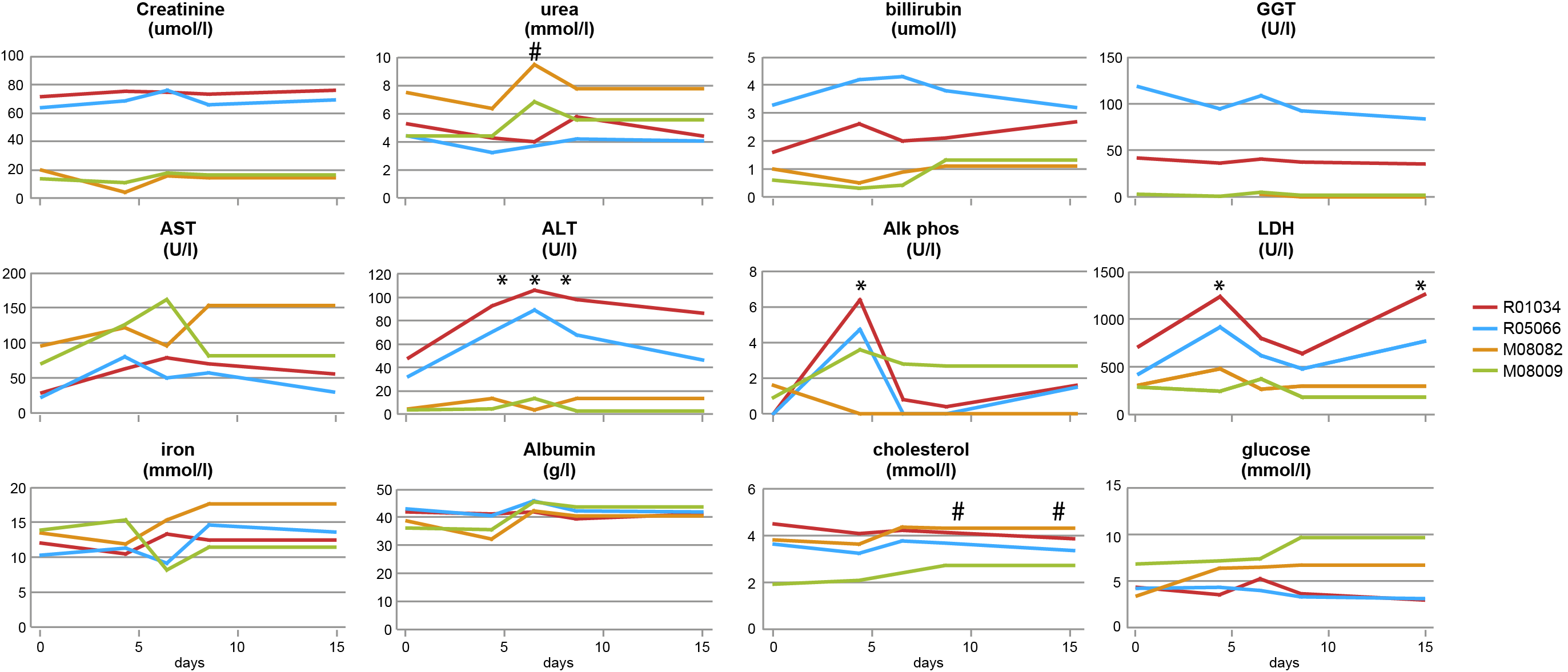

Supplement: Figure S1 — Serum chemistry data. Changes in biochemistry parameters of the individual rhesus macaques (red and blue lines) and common marmosets (orange and green lines) during the 14-days follow-up period. Day 0 indicates the day of WNV infection. The mean value of the two rhesus macaques measured at time point x was compared with the mean value of the two rhesus macaques at time point 0 using a 2-way ANOVA test. Statistical difference was defined as p<0.05 (represented by * for rhesus macaques). Same method was used to calculate statistical difference in common marmoset samples and this is represented by the # in graphs. (TIF) [file pntd.0002797.s001.tif]

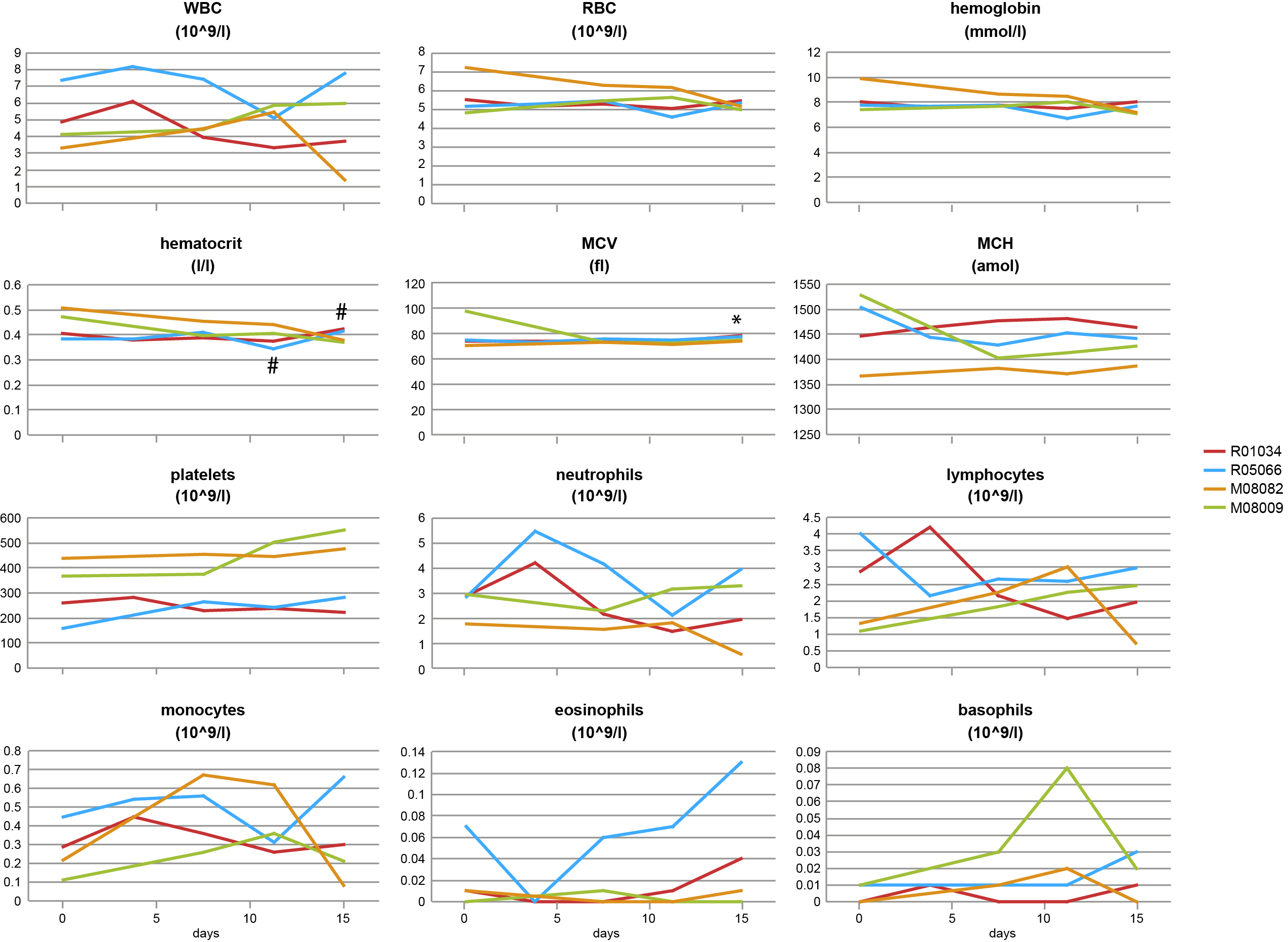

Supplement: Figure S2 — Hematology data. Changes in hematology parameters during the 14-days follow-up period of the in individual rhesus macaques (red and blue lines) and common marmosets (orange and green lines). Day 0 indicates the day of WNV infection. The mean value of the two rhesus macaques measured at time point x was compared with the mean value of the two rhesus macaques at time point 0 using a 2-way ANOVA test. Statistical difference was defined as p<0.05 (represented by * for rhesus macaques). Same method was used to calculate statistical difference in common marmoset samples and this is represented by the # in graphs. (TIF) [file pntd.0002797.s002.tif]

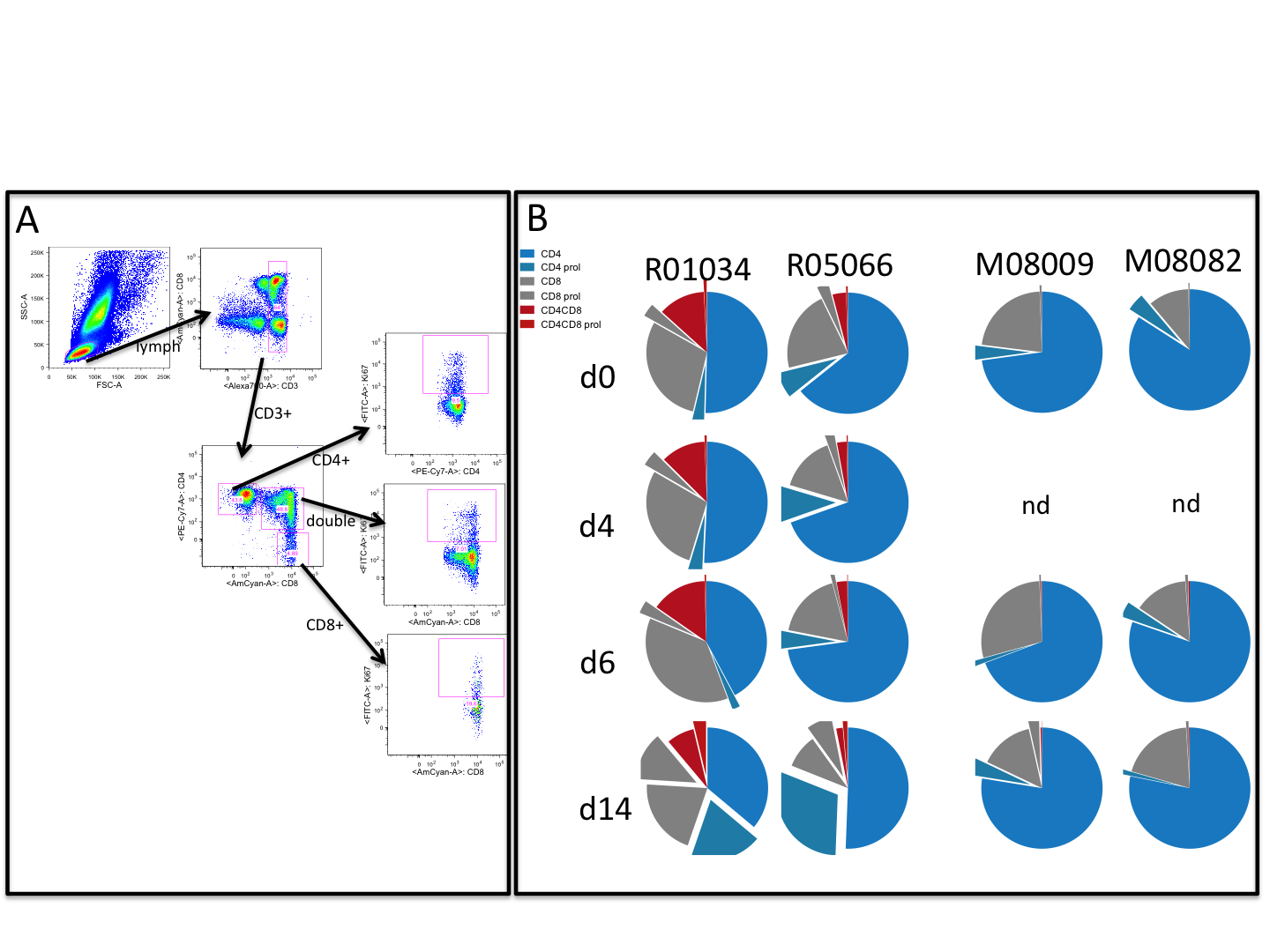

Supplement: Figure S3 — Proliferative T-cells in PBMC after WNV infection. (A) A representative example of the gating strategy. Cells in the lymphogate were selected based on the expression of CD3. CD3+ cells were divided into CD4+, CD8+, or CD4+CD8+ double-expressing T-cells. Next, Ki67 expression was determined as a measure for the proliferative properties of the different T-cell populations. (B) Full circle represents the total T-cell fraction per animal per time-point. Within this fraction the CD4+ (blue), CD8+ (grey), and CD4+CD8+ (red) T-cells are indicated. The exploded slices from the pie represent the share of proliferating cells per subtype. (TIFF) [file pntd.0002797.s003.tif]

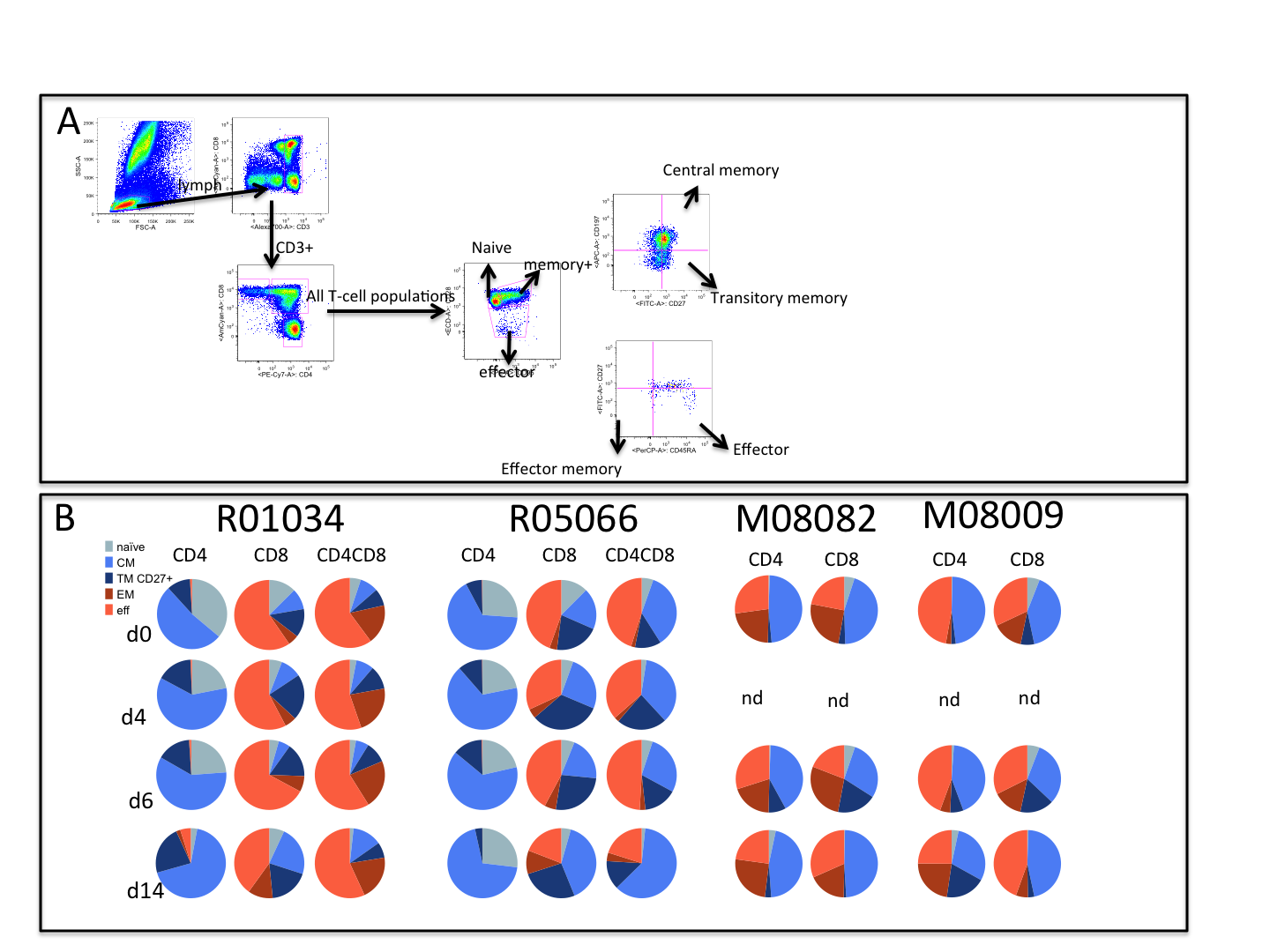

Supplement: Figure S4 — T-cell differentiation during WNV infection. (A) Representative example of the gating strategy. CD3 positive cells within the lymphogate were divided into three different populations based on the expression of CD4 and CD8. CD4 T-cells, CD8 T-cells, and double-positive T-cells were divided into naive (CD28+CD95−), memory (CD28+CD95+), and effector cells (CD28−). Next, based on the expression of CCR7 and CD27, memory cells were divided into central memory (CM) (CCR7+CD27+) and transitional memory (TM) (CCR7−CD27+) cells. Effector cells were analyzed for the expression of CD45RA, and split into effector memory cells (EM: CD45RA−) and effector cells (CD45RA+). (B) Pie diagrams of the differentiation status of CD4+, CD8+, and double-positive T-cells. Full circles represent the total CD4+, CD8+, or CD4+CD8+ T-cell populations in each animal at different time-points post-infection. (TIFF) [file pntd.0002797.s004.tif]

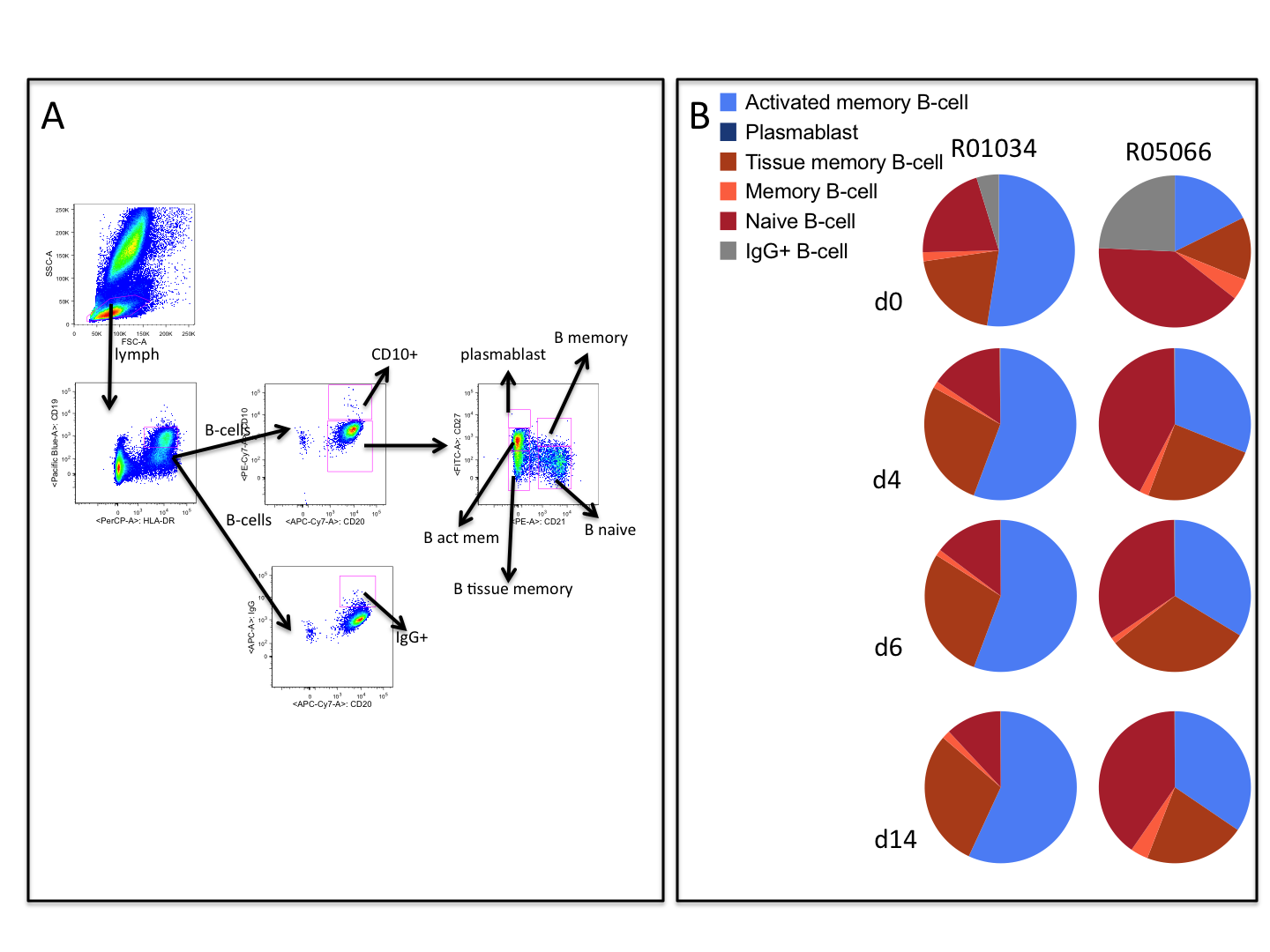

Supplement: Figure S5 — B-cell subsets during WNV infection in macaques. (A) Representative example of the gating strategy. CD19+HLA-DR+ cells were divided into CD10+ and CD10− cells. Next, based on the expression of CD27 and CD21, CD10 negative B-cells were divided into plasmablasts, memory B-cells, naive B-cells, activated memory B-cells, and tissue memory B-cells. The percentage of IgG-producing the total number of B-cells was analyzed. (B) The full circles represent the total number of B-cells of the individual animals at different time points while the pie parts represent different B-cell subsets. (TIFF) [file pntd.0002797.s005.tif]

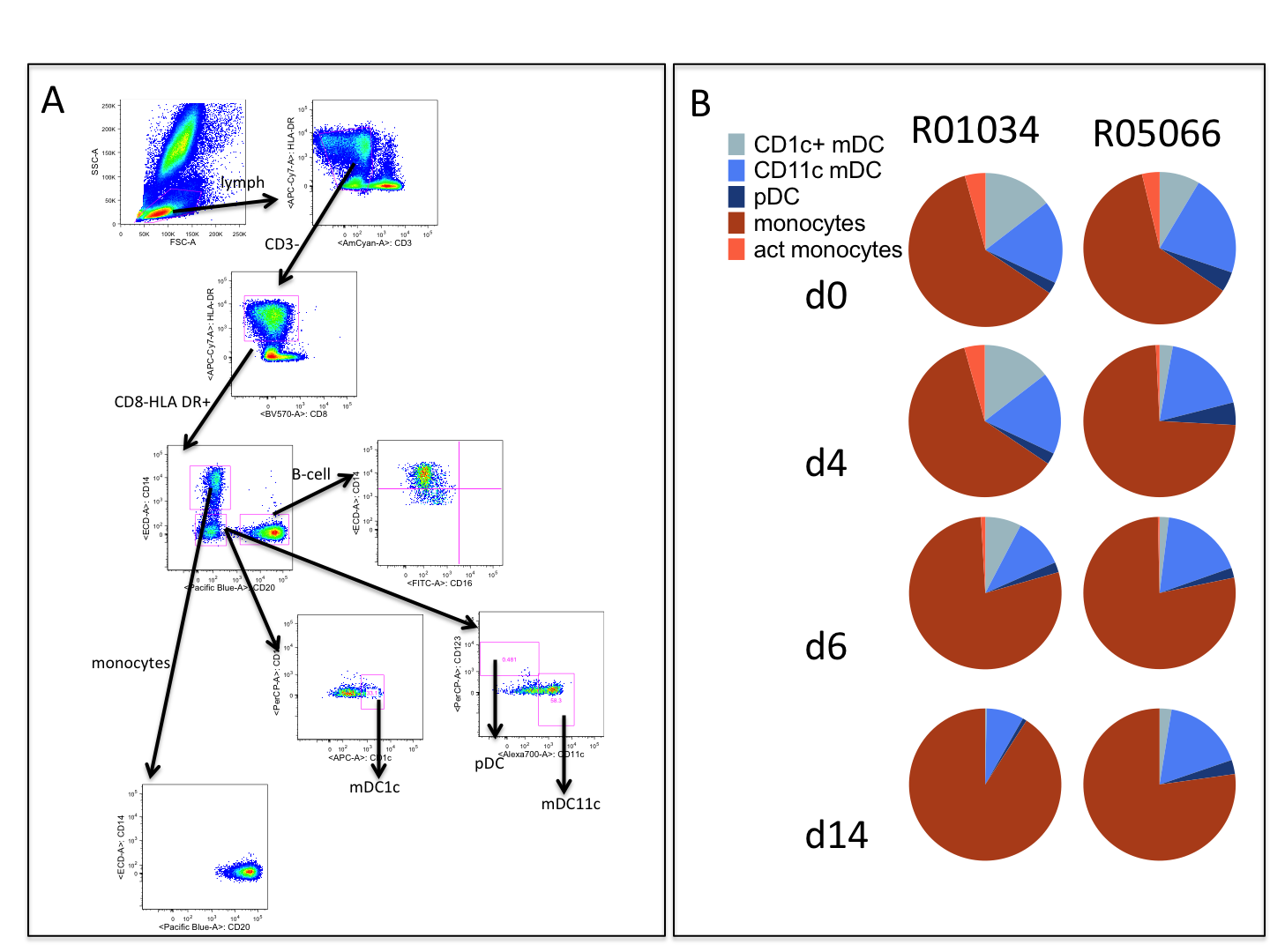

Supplement: Figure S6 — Dendritic cell subsets during WNV infection in rhesus macaques. (A) Representative example of the gating strategy for the determination of the different DC subsets. CD3−, CD8−, HLA-DR+ cells were selected from the lymphogate. Cells negative for CD20 were analyzed for the expression of CD14. CD14+ cells were defined as activated monocytes while CD14− cells were divided into CD1c mDC (CD123−CD1+), pDC (CD123+CD11−) and CD11c mDC (CD123−CD11+). (B) The full circles represent the total number of CD3−HLA-DR+D20− dendritic cells while the pie parts represent the different DC subsets at the different time points. (TIFF) [file pntd.0002797.s006.tif]
