# Supplementary material for: Experimental Infection of Rhesus Macaques and Common Marmosets with a European Strain of West Nile Virus
Source: PLoS Negl Trop Dis. 2014 Apr 17;8(4):e2797. doi: 10.1371/journal.pntd.0002797 (PMC3990483; doi:10.1371/journal.pntd.0002797)
Supplement: Table S1 — FACS antibodies used in study. List of antibodies used to assess changes in lymphocyte subset composition during WNV infection. (DOCX) [file pntd.0002797.s007.docx]

**Supplementary Table S1**

**FACS antibodies used in study**

| Tube | Name | Antibodies |
| --- | --- | --- |
| Rh1 | General lymphocyte panel | CD20-FitC (L27, Becton Dickinson)  CD4-PE-Cy7 (SK-3, Becton Dickinson)  CD8-V500 (SK-1, Becton Dickinson)  CD3-APC (SP34-2, Becton Dickinson)  CD14-PE-TxRed (RMO52, Beckman Coulter)  CD16-PE (3G8, Becton Dickinson)  HLA-DR-PerCP (L243, Becton Dickinson)  CD69APC-Cy7 (FN50, Becton Dickinson). |
| Rh2 | Memory and naive subsets | CD3-AF700 (SP34-2, Becton Dickinson)  CD4-PE-Cy7 (SK3, Becton Dickinson)  CD8-V500 (SK1, Becton Dickinson)  CD27-FitC (MT271, Becton Dickinson)  CD28-PE-TxR (28.2, Beckman Coulter)  CD95-PE (DX2, Becton Dickinson)  CD45RA-biotin (5H9; Becton Dickinson) CD197-APC (150503, R&D). |
| Rh3 | Regulatory T-cells | CD3-AF700 (SP34-2, Becton Dickinson)  CD4-PE-Cy7 (SK3, Becton Dickinson)  CD8-V500 (SK1, Becton Dickinson)  CD25-PE (4E3, Miltenyi)  CD28-PE-TxRed (CD28.2, Beckman Coulter)  CD45RA-biotin ( 5H9, Becton Dickinson)  Ki67-FitC (B56, Becton Dickinson)  CD152-APC (BN13, Becton Dickinson). |
| Rh4 | Dendritic cell subsets | CD3-AF700 (SP34-2, Becton Dickinson)  CD8-V500 (SK1, Becton Dickinson)  CD56-APC (AF12-7H3, Miltenyi)  CD14-PE-TxRed (RMO52, Beckman Coulter)  CD16-APC-Cy7 (3G8, Becton Dickinson)  CD20-V500 (L27, Becton Dickinson)  CD161-PE (DX12, Becton Dickinson)  CD45-FitC (MB4-6D6, Miltenyi)  CD336-PE-Cy5 (Z231, Beckman Coulter)  CD159a-PE-Cy7 (Z199, Beckman Coulter). |
| Rh5 | Dendritic cell subsets | CD3-V500 (SP34-2, Becton Dickinson)  CD4-PE-Cy7 (SK3, Becton Dickinson)  CD8-BrViolet (RPA-T8, Becton Dickinson)  CD20-V500 (L27, Becton Dickinson)  CD16-FitC (3G8, Becton Dickinson)  CD14-PE-TxRed (RMO52, Beckman Coulter)  HLA-DR-APC-Cy7 (L243, Becton Dickinson)  CD11c-AF700 (3.9, eBioScience)  CD1c-APC (L161, Miltenyi)  CD163-PE (GJI/61, Becton Dickinson) CD123-PerCP-Cy5.5(7G3, Becton Dickinson). |
| Rh6 | B-cell subsets | CD19-PB (J3.119, Beckman Coulter)  CD21-PE (BLy4, Becton Dickinson)  CD27-FitC (MT271, Becton Dickinson)  CD14-PE-TxRed (RMO52, Beckman Coulter)  HLA-DR-PerCP (L243, Becton Dickinson)  CD10-PE-Cy7 (HI10a, Becton Dickinson)  CD20–APC-Cy7 (L27, Becton Dickinson)  IgG-APC (G18-145, Becton Dickinson). |
| M1 | General lymphocyte panel | CD3-V500 (SP34-2, Becton Dickinson)  CD4-PE-CY7 (SK3, Becton Dickinson)  CD8-AF700 (LT8, Ab Serotec)  CD69-APC-CY7 (FN50, Becton Dickinson)  HLA-DR-PerCP (L243, Becton Dickinson)  CD14-PE-TxRed (RMO52, Coulter)  CD16-FitC (3G8, Becton Dickinson)  CD20-PE (H299, Coulter). |
| M2 | Memory and naive subsets | CD3-V500 (SP34-2, Becton Dickinson)  CD4-PE-CY7 (SK3, Becton Dickinson)  CD8-AF700 (LT8, AbSerotec)  CD28-PE-TxRed (CD28.2, Coulter)  CD45RA-biotin (5H9, Becton Dickinson)  CD197-APC (150503, R&D)  CD27-FitC (MT271, Becton Dickinson)  CD95-PE (DX2, Becton Dickinson) |
| M3 | Regulatory T-cells | CD3-V500 (SP34-2, Becton Dickinson)  CD4-PE-CY7 (SK3, Becton Dickinson)  CD8-AF700 (LT8, AbSerotec)  CD28-PE-TxRed (CD28.2, Coulter)  CD45RA-biotin (5H9, Becton Dickinson)  CD25-PE (4E3, Miltenyi)  Ki67-FitC (B56, Becton Dickinson)  CD152-APC (BN13, Becton Dickinson). |
| M4 | NK | CD3-V500 (SP34-2, Becton Dickinson)  CD8-AF700 (LT8, AbSerotec)  CD45-FitC (MB4-6D6, Miltenyi)  CD161-PE (HP-3G10, eBioscience)  CD14-PE-TxRed (RMO52, Coulter)  CD336-Pe-Cy5 (Z231, Coulter)  CD159a-PE-Cy7 (Z199, Beckman Coulter)  CD16-APC-Cy7 (3G8, Becton Dickinson) CD56-APC (AF12-7H3, Miltenyi) |
